# A computational and structural approach to identify malignant non-synonymous FOXM1 single nucleotide polymorphisms in triple-negative breast cancer

**DOI:** 10.1038/s41598-024-85100-w

**Published:** 2025-01-06

**Authors:** Prarthana Chatterjee, Satarupa Banerjee

**Affiliations:** School of BioSciences and Technology, Vellore Institute of Technology, Vellore, Tamil Nadu 632014 India

**Keywords:** Triple-negative breast cancer, FOXM1, Single nucleotide polymorphisms, Molecular dynamic simulations, Diagnostic markers, Breast cancer, Cancer genomics

## Abstract

The proliferation-specific oncogenic transcription factor, FOXM1 is overexpressed in primary and recurrent breast tumors across all breast cancer (BC) subtypes. Intriguingly, FOXM1 overexpression was found to be highest in Triple-negative breast cancer (TNBC), the most aggressive BC with the worst prognosis. However, FOXM1-mediated TNBC pathogenesis is not completely elucidated. Single nucleotide polymorphisms (SNPs) are the most common genetic variations causing functional and structural aberrations in proteins enhancing cancer susceptibility. This computational investigation attempted to identify the malignant FOXM1 non-synonymous SNPs (nsSNPs) and evaluate their role in affecting the conformational and functional stability, evolutionary conservation, post-translational modifications, and malignant susceptibility of the protein. Out of a huge data pool of 8826 FOXM1 SNPs using several in-silico sequence-based tools and structural approaches, four SNPs viz. E235Q, R256C, G429E and S756P were identified as pathogenic nsSNPs and among the shortlisted variants molecular dynamics simulations identified E235Q as the most damaging malignant SNP, followed by S756P. Additionally, the defective drug and DNA binding motif of E235Q and S756P were also determined in our study. Thus, although further in-vitro validations are awaited the findings of this in-silico work can be used as a blueprint for malignant nsSNP identification of FOXM1 aiding in clinical TNBC therapeutics.

**Supplementary Information:**

The online version contains supplementary material available at 10.1038/s41598-024-85100-w.

## Introduction

Contributing to about 15-20% of the invasive breast cancer (BC) burden reported annually worldwide, Triple-negative breast cancer (TNBC) is the most refractory and aggressive form of the disease^1–3^. Based on immunohistochemistry (IHC) TNBC lacks the expression of the three major hormone receptors, estrogen (ER), progesterone (PR) and the human epidermal growth factor receptor 2 (HER2)^4,5^. The unusual metastatic pattern, early onset at a younger age, rapid relapse rate, absence of targeted therapies and therapy resistance are the defining clinical characteristics of the disease^6,7^. Owing to the absence of hormone receptors, most conventional therapeutic paradigms targeting ER, PR and HER2 as in other BC subtypes are ineffective in impending TNBC progression^8^. Hence, the incidence, progression and therapy response in TNBC might not be restricted to any single etiological parameter but emanate from multiple genetic, epigenetic, lifestyle and ethnic factors. Even across individuals, there may be striking differences in the level of oncogenesis and curative sensitivity attributed to their genomic diversity^9–12^. Therefore comprehending the comprehensive tumor genetic landscape can facilitate the innovation and implementation of possibly efficacious and tailored personalized TNBC treatment strategies.

A member of the Forkhead/winged helix superfamily of transcription factor (TF), the proliferation-promoting oncogenic FOXM1 is found to be overexpressed in 85% of TNBC patients and has been reported as a top-ranked overall survival (OS)-related TF in TNBC^13,14^. The human Fox gene family consists of 40 subfamily members including FOXM1. The common feature of this subfamily of TFs is that they possess a 110 amino acid conserved DNA binding region with a “wing-like-helix” architecture^15,16^. The human FOXM1, also referred to as Trident is a member of this FOX superfamily of TFs. The gene encoding the TF has a length of 19.47 kb with 10 exons located on chromosome band 12p 13.3^17,18^. FOXM1 is a proliferation-specific and proficient cell-cycle regulator actively participating in the repair of damaged DNA, stem cell renewal, hindrance of apoptosis, cell-cycle progression, cellular growth and proliferation, tissue regeneration, inflammation, metabolism, metastasis and angiogenesis^16,19,20^. Elevated FOXM1 expression has been detected in several malignancies including that of the breast, gastric cancer, pancreatic cancer, ovarian cancer and colorectal cancer^17,21–24^. Studies suggest FOXM1 overexpression confers malignant characteristics to tumor cells and thus plays a pivotal role in tumorigenesis, regulating tumor incidence, invasion and metastasis. Several studies have revealed FOXM1 overexpression is found in different molecular subtypes of BC, including luminal, HER2-responsive and hormone-deprived TNBC participating in initiation, invasion, proliferation, epithelial-mesenchymal transition, metastasis, angiogenesis and therapy resistance in BC patients^25,26^. Although FOXM1 overexpression has been observed in all the BC subtypes, it is noteworthy to mention strongest overexpression of FOXM1 has been reported in the primary and recurrent tumors of TNBC^20,27,28^. Gene expression profiling revealed FOXM1 overexpression was minimal in both Luminal A/B subtypes of BC, slightly more in HER2 whereas, the level of FOXM1 was observed to be highest in TNBC^20^. Hence from the results of these studies, it can be inferred that the differential FOXM1 expression across the different BC subtypes may be associated with the endocrine status of ER, PR and HER2 since FOXM1 overexpression was found to be closely linked with the deprived levels of ER, PR and HER2 status in TNBC. Furthermore, studies also reveal FOXM1 overexpression in basal TNBC, was also associated with upregulated expression of FOXM1 target genes^20^. Enhanced FOXM1 expression thus demonstrates impressive prognostic ability in TNBC diagnosis^28,29^. Hence considering the aforementioned studies FOXM1 can be used as a prospective therapeutic and prognostic biomarker in TNBC^20^. However, the therapeutic potential of the FOXM1-associated pathway in TNBC is still not completely elucidated.

Since tumor-associated aberrations can either be somatic or germline, treating the condition thus requires a thorough understanding of the underlying tumor characteristics. Single nucleotide polymorphisms (SNPs) are the most common cosmopolitan genetic alterations known to exert an enhanced impact on the underlying genetic landscape of any disease compared to other types^30,31^. SNPs are responsible for accounting for more than 90% of sequence variants, with an occurrence frequency of more than 1% in the general population^32,33^. The missense or the non-synonymous SNPs (nsSNPs) by virtue of amino acid substitutions in the exonic protein coding region are capable of altering the structure or functions of proteins associated with several diseases and disorders including cancer^34,35^. Each gene however comprises an enormous number of SNPs making it difficult to identify a particular candidate deleterious SNP. This underlines the necessity of both clinical correlation and convergence on a subset of all potential SNPs, which may aid in the curation of a highly reliable SNP subset for a particular gene that may correspond to any disease phenotype. However, evaluating every SNP at the clinical level is a time-consuming and exhaustive procedure^36,37^. Currently, computational predictors are available to evaluate and forecast the effect of a single genetic variant as in an SNP on a disease. Herein, a detailed and extensive computational investigation has been carried out using several in-silico webservers and computational tools such as molecular docking and molecular dynamics simulation studies to identify the most detrimental malignant nsSNPs of FOXM1 genome, and evaluate their functional and structural impact on FOXM1 TF which might affect TNBC prognosis. Concisely, the findings of this computational study aim to serve as a reliable and complete blueprint for further in-vitro validation of the malignant pathogenic nsSNPs associated with FOXM1 TF through wet lab studies.

## Materials and methods

### SNP dataset retrieval

The SNPs of the FOXM1 TF along with their corresponding reference IDs and the amino acid substitution positions were collected from the dbSNP (https://www.ncbi.nih.gov/snp/*)* database of NCBI (National Centre for Biotechnology Information Website)^38^. The canonical FASTA sequence of the FOXM1 protein was retrieved from the UniProt database (https://www.uniprot.org/*)*, ID: 08050 ^39^. Only the non-synonymous SNPs were further used for the study.

### Detrimental nsSNPs prediction

The classification of nsSNPs into distinct benign and malignant types is an elaborate process. However, a plethora of sophisticated bioinformatics web servers have been established lately to determine the conformational and functional impact of these single nucleotide polymorphisms or SNPs in the proteins^37^. These computational web servers were used to predict the deleterious/ damaging nsSNPs located in the FOXM1 coding region. Since cross-analyzing the pathogenic variants obtained from multiple web-server algorithms enhances result reliability, five different webservers viz. SIFT^40^, PANTHER-PSEP^41^, SNAP^42^, PolyPhen-2^43^and PON-P2^44^ were utilized in our study to predict the harmful FOXM1 nsSNPs. The SIFT (Sorting Intolerant From Tolerant) webserver, being our first choice in SNP categorization, is used almost in all computational work for nsSNP characterization. SIFT identifies the malignant nsSNPs by utilizing and comparing the protein’s homologous sequence with the paralogous and orthologous protein sequences. The SIFT algorithm categorizes a variant to be benign with the tolerance index score > 0.05, whereas a SIFT score higher than 0.05 defines a variant as malignant^40^. The PANTHER-PSEP (Protein ANalysis THrough Evolutionary Relationships-Position Specific Evolutionary Preservation) database estimates the likelihood of functional impact a harmful coding SNP exerts on the protein, thereby categorizing SNPs either as benign or damaging on the basis of the Pdel (probability of deleterious effect) score. Additionally, SNAP is another neural network-driven SNP classifier, which renders a confidence score and thus accurately categorizes the mutations of the variants either as neutral or damaging and their functional impact on the proteins^41^. Based on the PSIC (position-specific independent count) scores, the PolyPhen-2 webserver evaluates the mutational impact of the nsSNP from both functional and structural aspects of the protein. It defines the nsSNPs as benign, probably, or possibly damaging based on the difference in the PSIC scores of the variants^43^. A reliable machine-learning-based server, PON-P2 classifies nsSNPs into neutral, unknown, or harmful variants using a random-forest-based scoring method^44^. Nevertheless, the nsSNP classification accuracy was further enhanced by comparing the results obtained from the different aforementioned webserver algorithms and filtering the only deleterious nsSNPs for further evaluation in the study.

### Disease-related nsSNP prediction

Two web-based computational tools viz. PhD-SNP and SNPs&GO were further used to predict the degree of disease association of the harmful FOXM1 nsSNPs. The SVM-based machine-learning algorithm of SNPs&GO predicts harmful disease-linked nsSNPs with an accuracy rate of 82%^45^. A tolerance score of ≥ 0.5 implies the clinical pathogenicity of the evaluated nsSNP. Correspondingly, another online webserver, PhD-SNP classifies the SNPs into disease-causing or neutral subtypes based on a reliability index score graded between 0 and 9^46^.

### Estimation of nsSNPs impact on protein structure and function

MutPred2 is a machine-learning-based computational web application used to predict the phenotypic and molecular impact of the pathogenic amino acid substitution variants on protein folding and function^47^. A putative p-value score of ≥ 0.5 is generally considered to be associated with an addition or deletion of 14 multiple biophysical features evaluated by the tool.

### Estimation of nsSNPs impact on protein stability

The amino acid substitution impact rendered by the non-synonymous pathogenic variants of the FOXM1 gene on the protein stability is predicted using two distinct webserver tools viz. MUpro and I-Mutant. I-Mutant adapts a support vector machine algorithm to detect any variation in the stable conformation of the protein. A variant resulting in reduced protein stability is generally defined as malignant. A preset static temperature of 25^◦^C and pH7 is maintained for all inputs provided. The free energy change, DDG of the wild and mutant variant of the protein estimates its stability. A DDG < 0 implies unstable conformation of the protein, whereas a DDG > 0 indicates protein stability^48^. Correspondingly, the SVM approach of MUpro predicts the single-site mutation impact on protein stability designated by a confidence score. A score < 0 denotes impaired protein stability^49^.

### Estimation of protein evolutionary conservation

The ConSurf database was used to predict the protein’s evolutionally conserved amino acid residue sites. Using the Bayesian computation method, the server estimates phylogenetic connectivities by evaluating closely related homologous sequences^50^. For every individual residue of the protein, the conservation degree is calculated on a 1–9 scale of three categories: residues with a conservation score ranging between 1 and 4: variable, residues ranging between 5 and 6: intermediates, and those ranging between 7 and 9 are considered to be highly conserved. Residue substitutions at the conserved sites of the protein denote malignancy. The FASTA sequence of the FOXM1 protein was inserted as the sequence of query to determine the probable conserved motifs in concordance with the conservation score.

### Post-translational modification (PTM) sites prediction

A plethora of biological activities including protein-protein interactions and modulation of signaling cascades are governed by the protein’s post-translational modifications (PTM)^51^. Mutation-induced orthosteric and allosteric shifts often lead to differences in structural stabilization and energy conformations of the proteins. Hence interpreting the structural variations encompassing the PTM sites is very important to decode their impact on protein folding. Methylation, ubiquitination, phosphorylation, palmitoylation, glycosylation, hydroxylation and acetylation are the major PTMs affecting various progression of diseases including malignancies. MusiteDeep, a deep-learning-based predictor of PTM sites in proteins was used to predict the impact of the identified high-risk SNPs on the PTM sites of the FOXM1 transcription factor^52^(TF).

### Preparation of native and mutant FOXM1 structures

The three-dimensional X-ray crystallographic structure of the DNA-bound FOXM1 transcription factor (TF) was retrieved from the Protein database bank (PDB) (https://www.rcsb.org/pdb) in PDB format, PDB ID: 3G73^53,54^. The resolution of the selected crystallographic structure was 2.21Å. BIOVIA Discovery Studio was used to obtain a cleaned structure to eliminate the additional heteroatoms, water molecules, nucleic acid coordinates, and unique side chains from the DNA-bound FOXM1 PDB^55^. The retrieved structure of the native FOXM1 was then modeled using the online available homology modeling server, SWISS-MODEL for further analysis^56^.

Substitution missense mutations are known to impact the overall structural and functional stability of native proteins. Hence, post the identification of the most detrimental disease-associated SNPs, mutations were manually induced in the processed FOXM1 wild-type TF structure using mutagenesis wizard in PyMol to derive the crystallographic mutant models of identified deleterious SNPs^57^. The modeled structures were then refined using Mod-Refiner and the conformational validation of the native and mutant FOXM1 PDBs was performed using PROCHECK (https://servicesn.mbi.ucla.edu/.

PROCHECK/)^58^. The three-dimensional structure of the native and mutant FOXM1 was finally visualized using BIOVIA Discovery Studio^55^.

### Molecular dynamic simulations (MDS) of native FOXM1 and SNPs

MDS is a computational technique used to assess the physical movement of the atoms confined within an unbound protein or a protein complex^59^. It captures the interactive momentum of the atomic particles over a period of time. In this study, the atomic-level variations of native FOXM1 and its most pathogenic identified SNPs were estimated by performing 100ns MDS analysis. The MD simulations were performed using the GROMACS-2023.1 software package (GNU, General Public License; http://www.gromacs.org*)* in Ubuntu version 22.04.2 LTS^60^. The forcefield selected for this study was CHARMM36^61^. The native FOXM1 and its pathogenic variants were solvated in a dodecahedral water box of 100 cubic dimensions containing transferable inter-molecular three-point TIP3P crystallographic water molecules. The ionization states of the constituting amino acids of the protein were standardized at a physiological pH7 with a constant periodic boundary implementation^62^. This was followed by the Monte-Carlo method of electrostatic neutralization of the molecular complexes involving the addition of K + and Cl^−^ ionic charges. 1000 kJ/mol.nm2 force constant was implemented to restrict the motion of heavier atoms necessary to pertain to the native conformational folds of the protein thus eliminating steric overlap. The steepest descent algorithm was used for system geometry optimization, which constituted the first stage of the MDS. Following this, the system geometry underwent a two-stage equilibration involving 100ps iterations at each stage. Using the Berendsen temperature coupling protocol, the NVT (a constant quantification of particles, system volume, and temperature) ensemble was maintained throughout the first stage of system equilibration^63^. The Parrinello-Rahman barostat approach, applied at 1 atm pressure and 303.15 K temperature retained at constant NPT (a constant quantification of particles, pressure and temperature) ensemble provided further guidance throughout the second system equilibration stage^64^. The wild-type and mutant FOXM1 variants were then each subjected to a 100 ns MDS at a fixed NPT ensemble. High-range electrostatic interaction forces were calculated using the Particle Mesh Ewald (PME) technique with a 0.1 nm grid spacing and a unit 4 interpolation order^65^. All of the covalent bond lengths were constrained using the linear constraint (LINCS) technique, involving a 2 fs integration step-up time^63^. With coordinate restoration occurring every 1 ps, applying the Verlet cut-off algorithm, many of the hydrophobic non-bonded interactions were truncated at 10 Å. The various GROMACS in-built utilities were implemented to assess the MD trajectories of the individual unbound and DNA-bound wild-type and mutant complexes. Root-mean-square deviation (RMSD), root-mean-square fluctuations (RMSF), the radius of gyration (Rg), solvent-accessible surface area (SASA), hydrogen-bond and total energy deviations were estimated to obtain deeper insights to understand the impact of the missense substitution variations on the structural conformations of native FOXM1. The various MD trajectories were visualized using VMD and the corresponding graphs were generated using XMGRACE.

### Molecular docking of FOXM1 variants with DNA and protein-DNA contact analysis

The binding association between receptors and their corresponding ligand molecules is determined using the in-silico approach of molecular docking. The native FOXM1 TF and the SNPs were docked with a 26 bp double-helix DNA using the online docking webserver, HDOCK developed and maintained by Huang Laboratory, Huazhong University of Science and Technology^66^. With the input data provided for both the receptor and ligand molecule (either in the form of amino acid FASTA sequence or PDB structures), HDOCK uses a hybrid approach of template-based and template-independent docking to automatically anticipate the interaction between receptor and the ligand molecule. The webserver implements an intrinsic scoring algorithm for protein-DNA docking and thus can be used to decipher the molecular complexities of protein-nucleic acid interactions, based on the estimated complex structure. In this study, the PDB files of both the wild-type and mutated FOXM1 TFs were provided as the input receptor file, whereas the PDB structure of the DNA was uploaded to the web server as the ligand. In both the wild and mutated models of FOXM1 TFs, the same molecule of DNA was used as the ligand^67^. The HDOCK docking results obtained were directly sent to the email address provided and were also available on the result webpage. Out of the 10 best-docked poses obtained for each protein-DNA complex, the one with the most favorable binding affinity was downloaded and subjected to protein-DNA contact analysis using NUCPLOT analysis to identify the amino acid residues of FOXM1 majorly interacting with their consensus DNA motif^68^.

### Molecular docking and simulation of FOXM1 variants with Olaparib

Protein-ligand molecular docking of native and mutant FOXM1 variants was performed with an FDA-approved TNBC chemotherapeutic inhibitor, Olaparib using AutoDockTools (ADT) 1.5.6 ^69^. As a control drug Olaparib was selected in this study, over the another FDA-approved TNBC PARP inhibitor Talazoparib, as the former possess a better safety profile with reduced risk of anemia and neutropenia, and improved progression-free survival in sub-group analysis of TNBC patients, compared to Talazoparib^70^. The FOXM1 variants selected as receptors and Olaparib their common ligand, were uploaded in the ADT interface. The root detection method calculates ligand torsion, with aromaticity criteria fixed at 7.5. Required polar and non-polar hydrogen units were added to the receptor systems, along with the computation of their respective Gasteiger and Kollman charges^70^. After initial optimizations of the docking parameters, the receptor and ligand files were saved in .pdbqt formats. The grid box was then set in such a manner that it accommodated the predicted catalytic pockets in the receptors. Following this grid maps were generated using Autogrid and then Autodock was run. In the entire process, the receptor models were held rigid, whereas the ligand was kept flexible. The receptor binding affinity of the ligands was calculated in terms of binding energy (Kcal/mol). The lower the binding energy, the greater is presumed to be its affinity for receptor binding. Finally, the stability of docked complexes as inferred from molecular docking was further validated by performing MDS to calculate their respective values of RMSD.

## Results

**Fig. 1:**
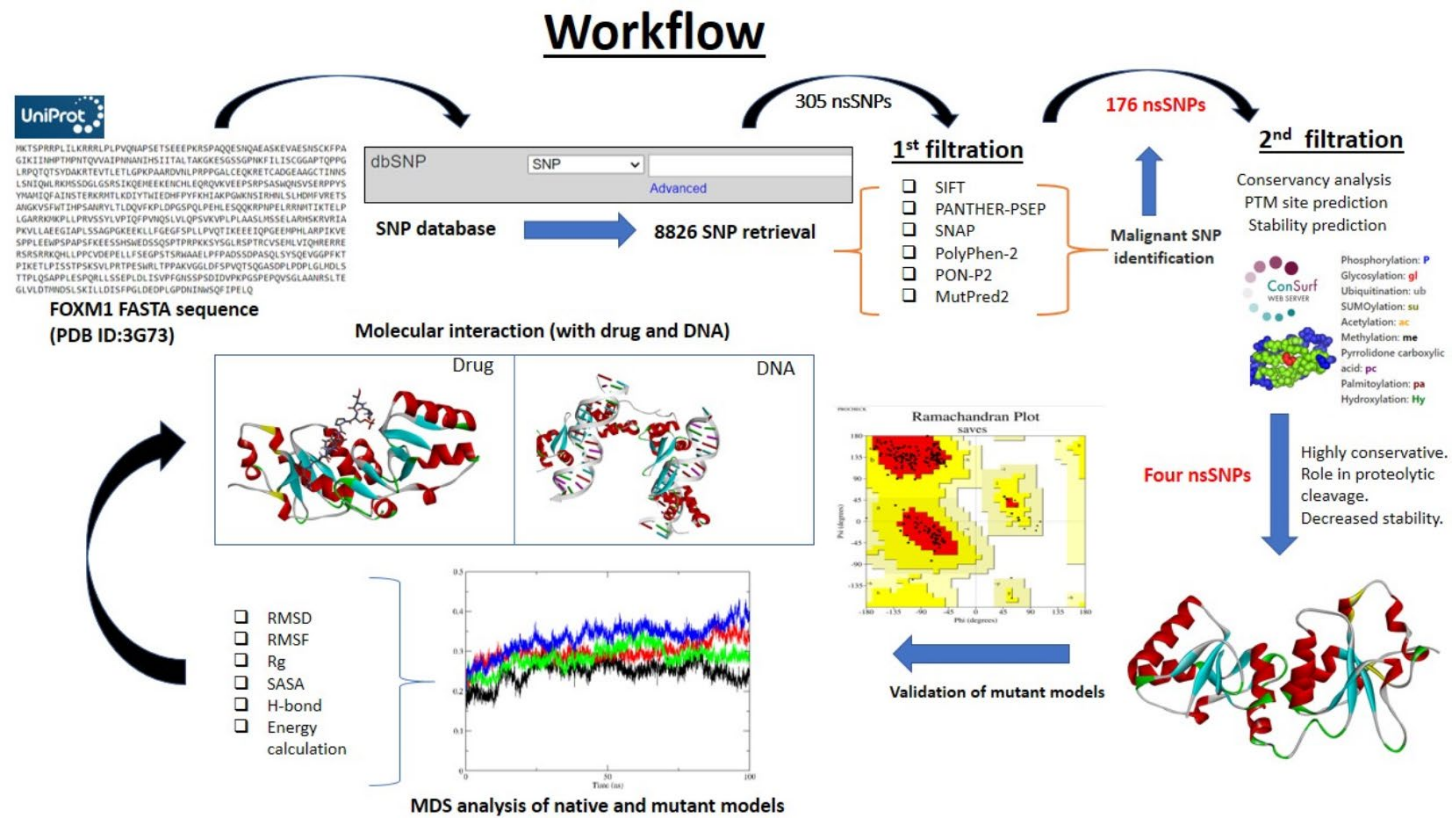
Detailed comprehensive workflow of the present computational study.

The extensive methodology and the webservers utilized in this study to identify the harmful and malignant nsSNPs of FOXM1 are depicted in Fig. 1.

### Analysis of SNP dataset retrieved

**Fig. 2:**
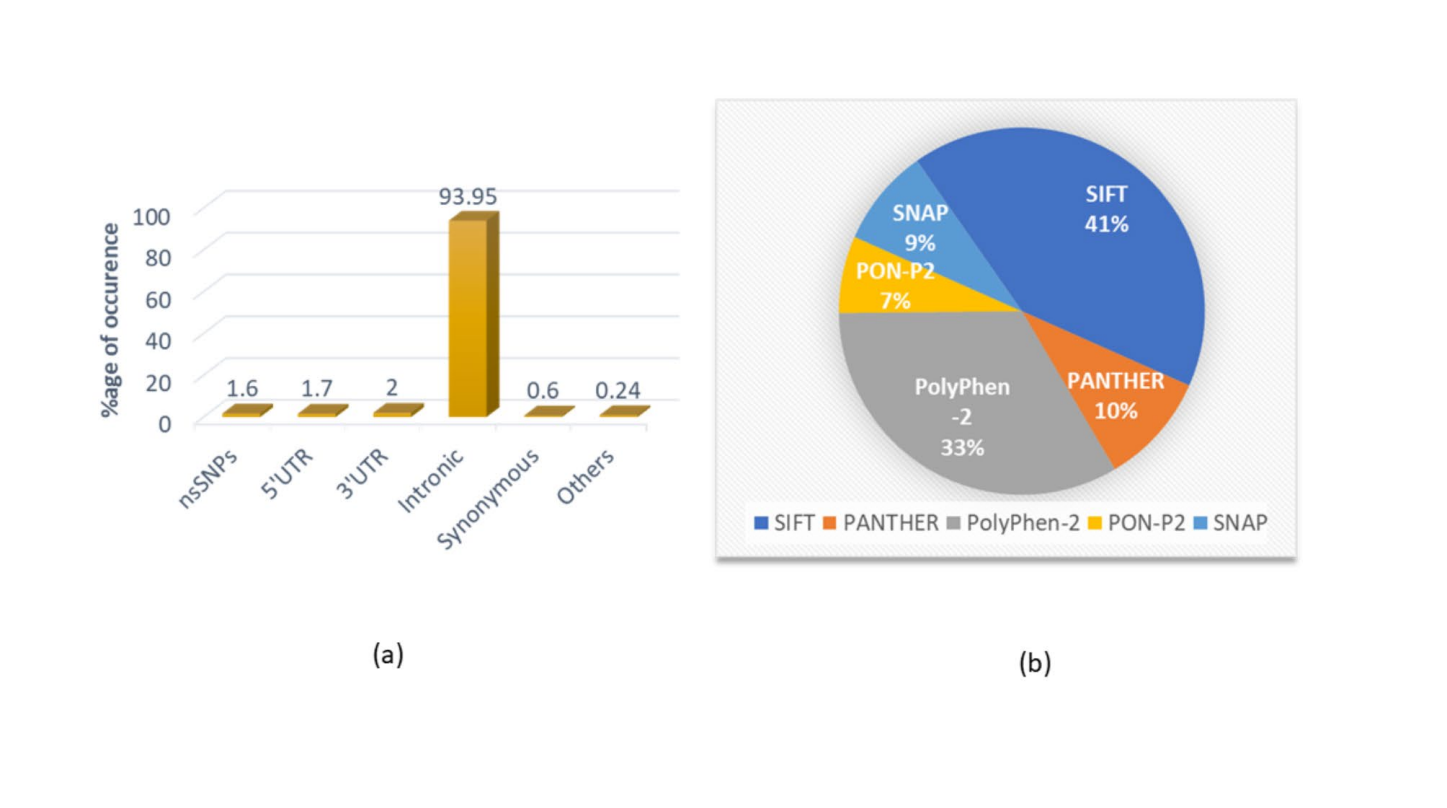
(**a** )Percentage of each SNP subtype of human FOXM1. (**b**) Percentage of high-risk nsSNPs of human FOXM1 predicted by different computational tools.

All the required information needed to understand the FOXM1 polymorphism was retrieved from the dbSNP database of NCBI. 8826 SNPs were reported from the dbSNP database about the FOXM1 transcription factor. Out of these 8826 SNPs, 7502 of them were found located in the intronic region, 297 SNPs were located in the 3’UTR, 200 SNPs were located in the 5’UTR, 142 were synonymous SNPs and 685 were non-synonymous SNPs. The percentage of each SNP subtype has been represented in Fig. 2a. In this study, we have only considered the nsSNPs for further analysis since missense mutation occurring at the nsSNPs is known to bring about a structural and functional alteration in the protein.

### Identification of detrimental nsSNPs

The high-risk or detrimental FOXM1 nsSNPs were identified using five different computational webservers viz. SIFT, PANTHER-PSEP, SNAP, PolyPhen-2, and PON-P2. The proportions of nsSNPs identified by these tools are depicted in Fig. 2b. SIFT identified 135 nsSNPs as malignant. The SIFT score ranges from 0 to 1. Any substitution variation with a score higher than 0.05 is considered malignant. PolyPhen-2 on the otherhand predicts the impact of amino acid substitution on the structure and function of the protein, with a score ranging from 0 to 1. A PolyPhen-2 score greater than 0.9 is interpreted as “probably damaging”, a score ranging between 0.446 and 0.908 is considered to be “possibly damaging”., while any score lesser than or equal to 0.446 corresponds to a benign SNP. The algorithm thereby anticipated 115 nsSNPs as detrimental. Additionally, PANTHER-PSEP is a tool which uses an empirical investigation of performance on a curated collection of known deleterious and neutral variations to estimate the probability of deleterious impact (P deleterious) from the preservation time. The PANTHER-PSEP programme has recognized 110 nsSNPs as non-benign. SNAP (screening for non-acceptable polymorphisms) is a neural-network based technique often used for predicting the functional implications of nsSNPs. It relies on a confidence score that depicts the likelihood that the anticipated effect on the protein function is correct. A high confidence score ranging from 0.9 to 1 implies considerable detrimental impact on protein function, whereas a low confidence score ranging between 0.0 and 0.49 implies low likelihood of accurate prediction on protein function. Both SNAP and PON-P2 identified about 105 and 96 damaging nsSNPs each. A total of 561 pathogenic missense mutations were detected using these five aforementioned web servers. Among these 561 missense nsSNPs detected, 76 nsSNPs were characterized as highly damaging by all of the five web servers and hence were prioritized further in the study (Table 1).

**Table 1:** Highlights the high-risk 76 nsSNPs of FOXM1 commonly identified by the five computational web-servers.

rsID	Substitution position	SIFT	PANTHER-PSEP	SNAP	PolyPhen-2	PON-P2
rs137928577	R256C	Deleterious	Probably damaging	Damaging	Probably damaging	Pathogenic
rs138730141	R717L	Deleterious	Probably damaging	Damaging	Probably damaging	Pathogenic
rs2098104940	Q443P	Deleterious	Probably damaging	Damaging	Probably damaging	Pathogenic
rs2098127983	V219D	Deleterious	Probably damaging	Damaging	Probably damaging	Pathogenic
rs2153931144	D684H	Deleterious	Probably damaging	Damaging	Probably damaging	Pathogenic
rs2153935069	M3671	Deleterious	Probably damaging	Damaging	Probably damaging	Pathogenic
rs2153935037	V373I	Deleterious	Probably damaging	Damaging	Probably damaging	Pathogenic
rs1216720660	S756P	Deleterious	Probably damaging	Damaging	Probably damaging	Pathogenic
rs1285576061	G429E	Deleterious	Probably damaging	Damaging	Probably damaging	Pathogenic
rs1478305288	L670V	Deleterious	Probably damaging	Damaging	Probably damaging	Pathogenic
rs1237999587	E483G	Deleterious	Probably damaging	Damaging	Probably damaging	Pathogenic
rs900046597	P331L	Deleterious	Probably damaging	Damaging	Probably damaging	Pathogenic
rs912730887	R570P	Deleterious	Probably damaging	Damaging	Probably damaging	Pathogenic
rs375005500	P475H	Deleterious	Probably damaging	Damaging	Probably damaging	Pathogenic
rs776324631	E235Q	Deleterious	Probably damaging	Damaging	Probably damaging	Pathogenic
rs763027795	L327M	Deleterious	Probably damaging	Damaging	Probably damaging	Pathogenic
rs762144757	Q645R	Deleterious	Probably damaging	Damaging	Probably damaging	Pathogenic
rs759395296	T510N	Deleterious	Probably damaging	Damaging	Probably damaging	Pathogenic
rs756264779	R256H	Deleterious	Probably damaging	Damaging	Probably damaging	Pathogenic
rs758750879	V407M	Deleterious	Probably damaging	Damaging	Probably damaging	Pathogenic
rs546655855	K462I	Deleterious	Probably damaging	Damaging	Probably damaging	Pathogenic
rs143720765	R164W	Deleterious	Probably damaging	Damaging	Probably damaging	Pathogenic
rs145011914	E721G	Deleterious	Probably damaging	Damaging	Probably damaging	Pathogenic
rs145831539	R546W	Deleterious	Probably damaging	Damaging	Probably damaging	Pathogenic
rs145537744	E470K	Deleterious	Probably damaging	Damaging	Probably damaging	Pathogenic
rs376160082	E357K	Deleterious	Probably damaging	Damaging	Probably damaging	Pathogenic
rs372363265	N751K	Deleterious	Probably damaging	Damaging	Probably damaging	Pathogenic
rs200595908	I78F	Deleterious	Probably damaging	Damaging	Probably damaging	Pathogenic
rs758750879	V408M	Deleterious	Probably damaging	Damaging	Probably damaging	Pathogenic
rs748086049	Y376C	Deleterious	Probably damaging	Damaging	Probably damaging	Pathogenic
rs569199069	P422R	Deleterious	Probably damaging	Damaging	Probably damaging	Pathogenic
rs369897716	T599I	Deleterious	Probably damaging	Damaging	Probably damaging	Pathogenic
rs369843426	R417H	Deleterious	Probably damaging	Damaging	Probably damaging	Pathogenic
rs754756552	G399R	Deleterious	Probably damaging	Damaging	Probably damaging	Pathogenic
rs758449989	R521G	Deleterious	Probably damaging	Damaging	Probably damaging	Pathogenic
rs139435894	P432R	Deleterious	Probably damaging	Damaging	Probably damaging	Pathogenic
rs139435894	R412G	Deleterious	Probably damaging	Damaging	Probably damaging	Pathogenic
rs138730141	S717L	Deleterious	Probably damaging	Damaging	Probably damaging	Pathogenic
rs146806643	R717H	Deleterious	Probably damaging	Damaging	Probably damaging	Pathogenic
rs150024483	P385L	Deleterious	Probably damaging	Damaging	Probably damaging	Pathogenic
rs759192397	P600H	Deleterious	Probably damaging	Damaging	Probably damaging	Pathogenic
rs759395296	T494N	Deleterious	Probably damaging	Damaging	Probably damaging	Pathogenic
rs765408731	N177S	Deleterious	Probably damaging	Damaging	Probably damaging	Pathogenic
rs767233350	P587T	Deleterious	Probably damaging	Damaging	Probably damaging	Pathogenic
rs767659718	T116N	Deleterious	Probably damaging	Damaging	Probably damaging	Pathogenic
rs2098119534	P372L	Deleterious	Probably damaging	Damaging	Probably damaging	Pathogenic
rs1170788329	S467G	Deleterious	Probably damaging	Damaging	Probably damaging	Pathogenic
rs1176724254	A227T	Deleterious	Probably damaging	Damaging	Probably damaging	Pathogenic
rs926667225	S685C	Deleterious	Probably damaging	Damaging	Probably damaging	Pathogenic
rs926667225	S646C	Deleterious	Probably damaging	Damaging	Probably damaging	Pathogenic
rs964344748	S717R	Deleterious	Probably damaging	Damaging	Probably damaging	Pathogenic
rs1182246756	K201N	Deleterious	Probably damaging	Damaging	Probably damaging	Pathogenic
rs1192802207	E363Q	Deleterious	Probably damaging	Damaging	Probably damaging	Pathogenic
rs995881166	R659S	Deleterious	Probably damaging	Damaging	Probably damaging	Pathogenic
rs971652336	E97Q	Deleterious	Probably damaging	Damaging	Probably damaging	Pathogenic
rs926667225	S648C	Deleterious	Probably damaging	Damaging	Probably damaging	Pathogenic
rs926667225	S647C	Deleterious	Probably damaging	Damaging	Probably damaging	Pathogenic
rs778570959	D737G	Deleterious	Probably damaging	Damaging	Probably damaging	Pathogenic
rs780171058	L515F	Deleterious	Probably damaging	Damaging	Probably damaging	Pathogenic
rs150152924	P329Q	Deleterious	Probably damaging	Damaging	Probably damaging	Pathogenic
rs1202581103	D642H	Deleterious	Probably damaging	Damaging	Probably damaging	Pathogenic
rs900046597	P344Q	Deleterious	Probably damaging	Damaging	Probably damaging	Pathogenic
rs775996793	R550G	Deleterious	Probably damaging	Damaging	Probably damaging	Pathogenic
rs773390706	V708A	Deleterious	Probably damaging	Damaging	Probably damaging	Pathogenic
rs774455070	T449N	Deleterious	Probably damaging	Damaging	Probably damaging	Pathogenic
rs773390706	V708A	Deleterious	Probably damaging	Damaging	Probably damaging	Pathogenic
rs770475263	T390A	Deleterious	Probably damaging	Damaging	Probably damaging	Pathogenic
rs759192397	P602H	Deleterious	Probably damaging	Damaging	Probably damaging	Pathogenic
rs749328694	V428M	Deleterious	Probably damaging	Damaging	Probably damaging	Pathogenic
rs200806960	P691L	Deleterious	Probably damaging	Damaging	Probably damaging	Pathogenic
rs749328694	V430M	Deleterious	Probably damaging	Damaging	Probably damaging	Pathogenic
rs569199069	P423R	Deleterious	Probably damaging	Damaging	Probably damaging	Pathogenic
rs376160082	E342K	Deleterious	Probably damaging	Damaging	Probably damaging	Pathogenic
rs201705172	P431R	Deleterious	Probably damaging	Damaging	Probably damaging	Pathogenic
rs200286055	R335W	Deleterious	Probably damaging	Damaging	Probably damaging	Pathogenic
rs749328694	V414M	Deleterious	Probably damaging	Damaging	Probably damaging	Pathogenic
rs151053319	R526W	Deleterious	Probably damaging	Damaging	Probably damaging	Pathogenic

### Identification of disease-related nsSNPs

The two web-based computational algorithms viz. PhD-SNP and SNPs&GO were then used to denote the degree of pathogenicity associated with these malignant FOXM1 nsSNPs. PhD-SNP is a SVM based predictor of deleterious human SNPs. It also relies on a confidence score range of 0 to 1. A high confidence score signifying higher likelihood of correct prediction, implies a damaging SNP, whereas any score ranging between 0.0 and 0.39 implies the change in the substitution variation to be less damaging or benign with respect to protein function. Correspondingly, SNPs&GO tool follows a reliability index (RI) that represents reliability of a prediction, ranging between 0 (unreliable) to 1 (reliable). A high RI score corresponds to a damaging SNP variant. Among 76 commonly denoted high confidence missense nsSNPs, 22 and 16 pathogenic disease-causing FOXM1 nsSNPs were identified by PhD-SNP and SNPs&GO. Finally among the 38 disease-causing pathogenic nsSNPs, four nsSNPs viz. E235Q, R256C, G429E and S756P were commonly identified by both tools, which were then subjected to further critical analysis (Table 2).

**Table 2:** Disease-associated shortlisted high-risk pathogenic nsSNPs of FOXM1.

rsID	Substitution position	PhD-SNP	SNPs&GO
rs776324631	E235Q	Diseased	Diseased
rs137928577	R256C	Diseased	Diseased
rs1285576061	G429E	Diseased	Diseased
rs1216720660	S756P	Diseased	Diseased

### Impact of nsSNPs on protein structure and functions

The structural and functional impact of the four short-listed pathogenic malignant FOXM1 nsSNPs were further evaluated using the online available computational server, MutPred2. Predicted alterations in the conformational and functional characteristics of FOXM1 pathogenic variants included loss of intrinsic disorder, disordered interface alteration, allosteric site loss, and N-linked glycosylation. The calculated values of the MutPred2 score and their impact on the structural and functional attributes of the FOXM1 transcription factor have been depicted in Supplementary Table 1. The MutPred2 score ranges from 0 to 1. A MutPred2 score ranging between 0 and 0.7 is generally considered to be high and extremely pathogenic. The highest MutPred2 score indicating the enhanced pathogenicity was reported in variants, E235Q (0.792) and S756P (0.739) respectively.

### Impact of nsSNPs on protein stability

The impact of missense substitution mutations on protein stability is computationally estimated using two web servers viz. Mupro and I-Mutant 2.0. When subjected to missense mutations, a negative value of free energy change designated by DDG indicates decreased and impaired protein stability, whereas a positive value of DDG implies enhanced protein stability. The corresponding values of DDG of the four most detrimental disease-associated nsSNPs of FOXM1 TF are tabulated in Table 3. A negative value of DDG in all four shortlisted malignant nsSNPs thus estimates a decrease in the overall stability of the FOXM1 protein.

**Table 3:** Impact of malignant nsSNPs on FOXM1. DDG, unfolding free energy change.

rsID	Substitution position	Mu-pro		I-Mutant	
		Prediction	DDG value (kcal/mol)	Prediction	DDG value (kcal/mol)
rs776324631	E235Q	Decreased	-2.05116	Decreased	-2.95
rs137928577	R256C	Decreased	-0.73523	Decreased	-1.84
rs1285576061	G429E	Decreased	-0.06331	Decreased	-1.71
rs1216720660	S756P	Decreased	-1.61611	Decreased	-2.13

### Analysis of protein evolutionary conservation

The amino acid sequence conservation analysis of the identified FOXM1 nsSNPs was performed using the ConSurf database. The highly conserved residues of a protein are known to affect its structural and functional attributes. At a range of 1–9, a confidence score of one indicated least conservation, whereas, a residue with a confidence score of 9 is considered highly conserved. All four non-synonymous FOXM1 variants possessed a conservation score of 9. The conserved buried residues as in E235Q and S756P are found to alter the structure of the protein, whereas exposed residues as observed in variants R256C and G429E are known to alter protein function (Table 4).

### PTM sites prediction

MusiteDeep was used to predict any PTM site changes in the four malignant altered residues of FOXM1 TF viz. E235Q, R256C, G429E and S756P. Of these four nSNPs, R256C and S756P were located at a glycosylated site. However, E235Q and G429E were found to have no role in the PTM modification of the TF.

**Table 4:** Evolutionary conservation profile and PTM site prediction of the four high-risk malignant FOXM1 nsSNPs.

rsID	Substitution position	Conservation score	Prediction	PTMs
rs776324631	E235Q	9	Conserved and buried (s)	-
rs137928577	R256C	9	Conserved and exposed (f)	Glycosylated
rs1285576061	G429E	9	Conserved and exposed (f)	-
rs1216720660	S756P	9	Conserved and buried (s)	Glycosylated

### Native and mutant FOXM1 structure preparation

The FOXM1 PDB ID: 3G73 is the best available DNA-bound structure of the TF. The protein was further modeled using a homology-modeling server, SWISS-MODEL, and the substitution mutations were introduced using the mutagenesis wizard in PyMol. The structures of the modeled proteins were further validated using the Ramachandran plot analysis of the PROCHECK plugin, SAVES 6.0 version (https://saves.mbi.ucla.edu/) (Supplementary Fig. 1). The validated structures of the native and mutant models were eventually subjected to molecular dynamics simulation and protein-ligand interaction analysis.

### MDS analysis of FOXM1 variants

**Fig. 3:**
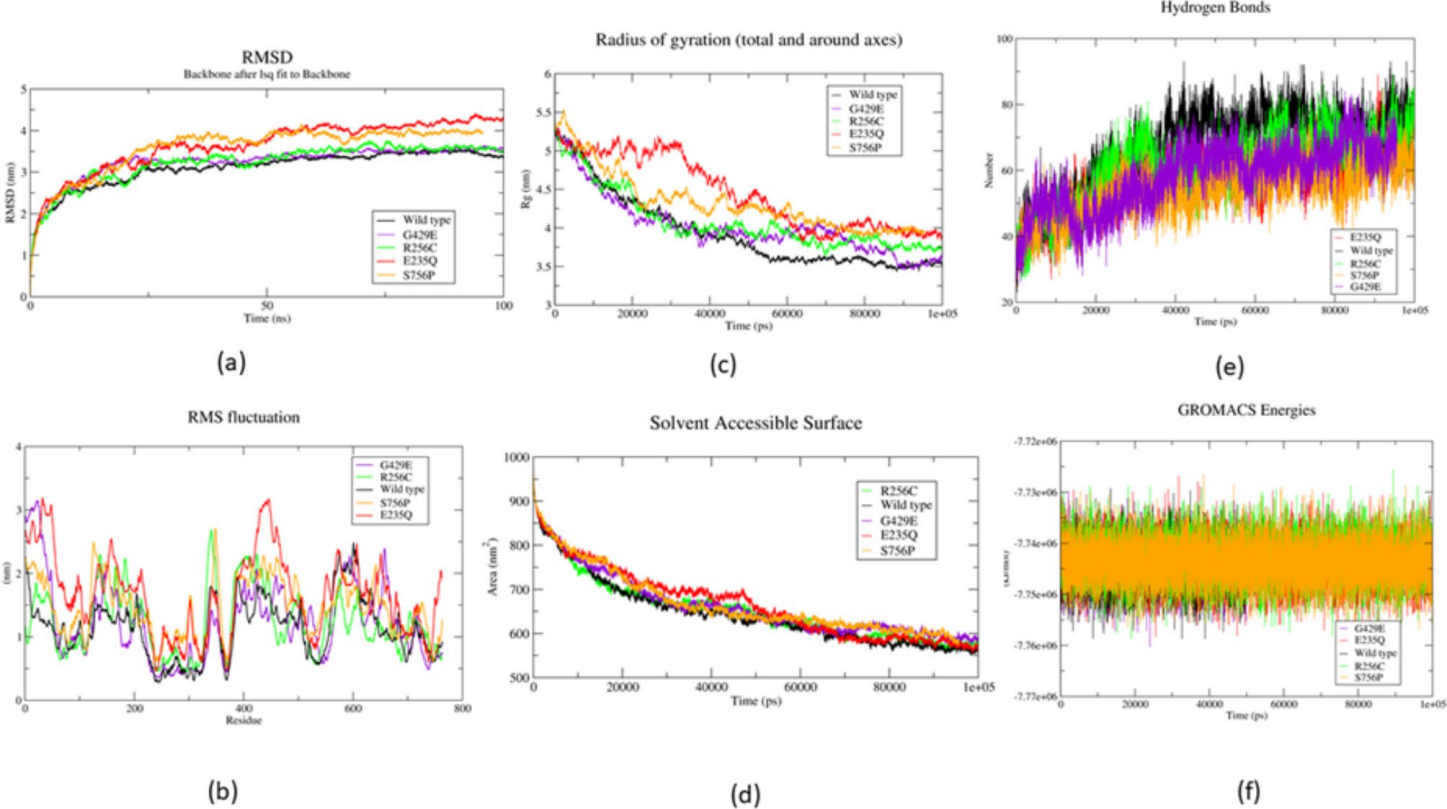
MDS run of 100ns wildtype FOXM1 and mutants depicting (**a**) RMSD (**b**) RMSF (**c**) Rg (**d**) SASA (**e**) H-bond (**f**) Total energy.

MDS is extensively employed to understand the dynamic changes in the structural conformation and function of biomolecules when subjected to frequent molecular adversities such as mutations. 100ns MD simulation was carried out individually for native FOXM1 and its variant models E235Q, R256C, G429E and S756P. The backbone and the mechanistic stability of the wild and mutant protein systems are estimated by calculating their respective RMSD values, using the crystal structures of TF as reference and Cα atoms for least fitting. From the combined RMSD graph of the native and mutant FOXM1 models (Fig. 3a), it is observed that the lowest mean RMSD value was recorded for the wild-type native model of FOXM1 (3.103631 nm), followed by intermediate RMSD values for SNPs R256C (3.231345 nm) and G429E (3.247829 nm) respectively. Contrastingly, higher values of mean RMSD were observed for S756P and E235Q corresponding to 3.57209 nm and 3.64377 nm respectively. The least mean RMSD value of the wild FOXM1 system compared to its shortlisted deleterious mutants, denotes higher equilibrium conformation of the protein with least atomistic backbone deviations and enhanced stability. Significant deviations in RMSD values of FOXM1 SNPs E235Q and S756P signify a lack of structural integrity and enhanced conformational unrest when subjected to missense mutations contributing to its malignant transformation. For obtaining better and more valuable insights regarding the conformational behaviour of the four mutants viz. E235Q (denoted in red), S756P (denoted in orange), R256C (denoted in green), G429E (magenta) and the wild-type FOXM1 (denoted in black), we further extended the MD simulation to 200ns, and the combined RMSD graph of the mutants and the wild-type FOXM1 have been provided in Supplementary Fig. 2. No significant changes in the RMSD trajectory were observed with respect to the behaviour of the mutants and the wild-type. E235Q followed by S756P observed the highest values of RMSD over 200ns MD run- 4.0872 nm (E235Q) and 3.8963 nm (S756P). The least RMSD value of 3.1002 nm was retained for the wild-type native FOXM1 TF.

To investigate, the dynamic conformational perturbations of the native and mutant FOXM1 variants, RMSF was calculated which essentially mimics the Cα atom mobility of the constituting amino acid residues to decipher the residue-level contribution to backbone flexibilities. Figure 3b depicts the combined RMSF graph of wild and mutant variants exhibiting the amplitude of their atomic displacements. A higher value of mean RMSF indicates greater backbone flexibility resulting in enhanced residue-level fluctuations resulting in eventual conformational and functional assault of the protein. It can be observed from Fig. 3b, the E235Q variant of FOXM1 TF has a greater number of higher fluctuating peaks (denoted in red) with the maximum value of average RMSF, 1.66501 nm, followed by the variant S756P (denoted in orange), where the calculated mean RMSF value is 1.46407 nm. In concordance, with the value of RMSD, the least mean RMSF (1.13842) was observed in the wild-type FOXM1 model (indicated in black) with, shorter fluctuating peaks reflecting the stable and minimum flexibility of the native system.

Rg gives a further estimate of the total protein dimension and measures the overall compactness of the native and mutant variants. Rg also demonstrates the consistent and close packaging of the secondary structure elements of the protein into a complex three-dimensional structure. The average Rg of the native and mutants were found to range between 3.94575 and 4.45637 nm (Fig. 3c). The highest average Rg was observed in the mutant E235Q (4.45637 nm) implying considerable conformational distortion and complex instability, followed by variant S756P (4.33733 nm). Interestingly, the mean Rg value of the G429E variant was observed to be the least among all the four mutants (4.02269 nm), with the wild-type FOXM1 protein, exhibiting the least mean calculated Rg of 3.94575 nm. Additionally, SASA was calculated to estimate the protein’s affinity towards solvent interactions. It was observed from the combined SASA graph of wild-type and FOXM1 mutant models (Fig. 3d) that the four shortlisted malignant SNPs of FOXM1 exhibited higher and inconsistent SASA values with the native FOXM1 structure each evaluated over 100ns MD simulation. In accordance with the results of Rg, the highest and least SASA values were observed in the variant E235Q and wild-type FOXM1 respectively. The significantly higher SASA value of E235Q (670.1707 nm^2^) and S756P (667.066 nm^2^) variants than the wild type (646.1153 nm^2^) FOXM1 TF, is thereby suggestive of an erratic shift in the hydrophobic contact among the constituent amino acid residues of the protein from the hydrophilic zone to the buried region of the mutants relative to the wild type.

**Fig. 4:**
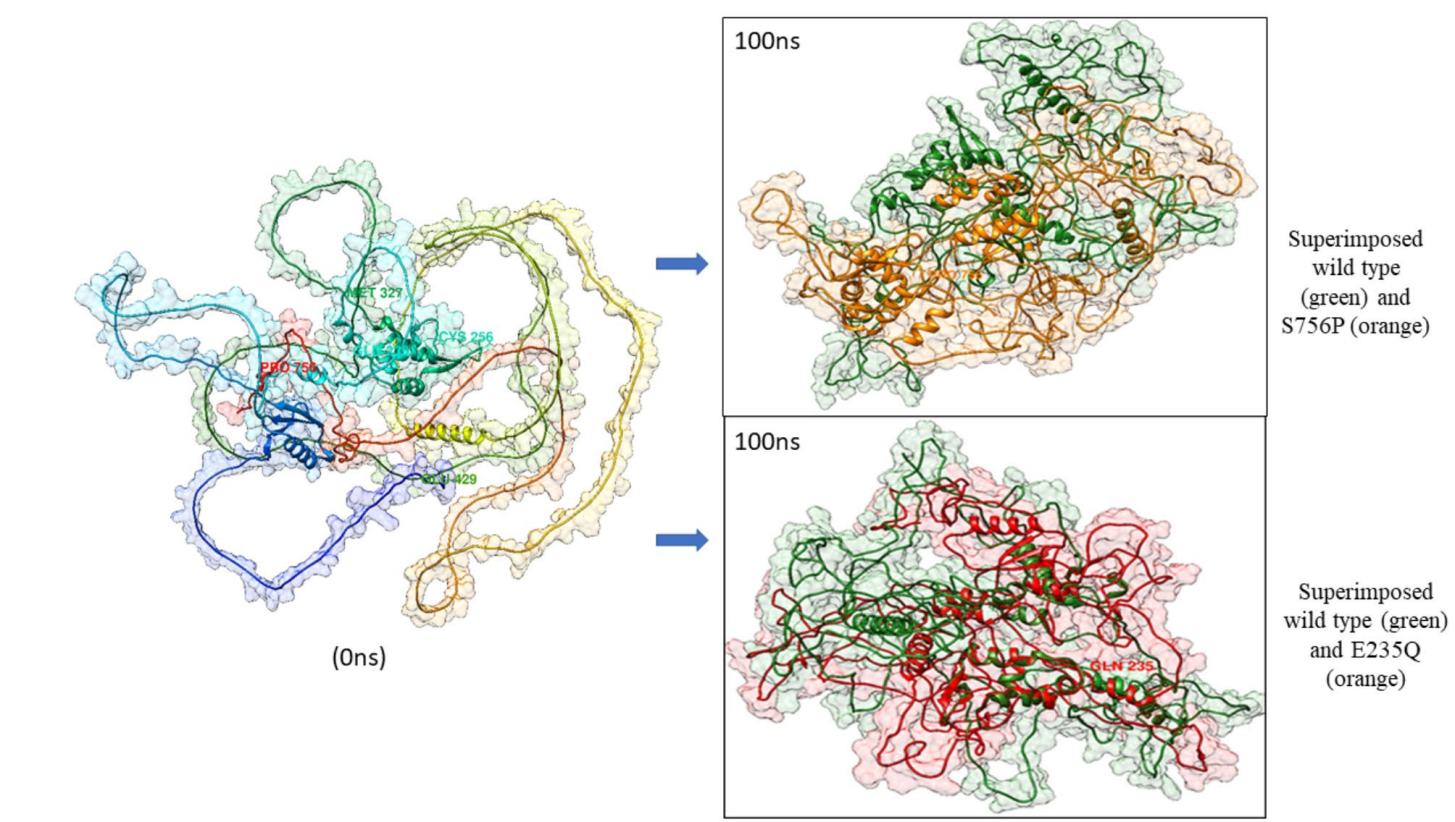
Superimposed images of human FOXM1 variants S756P and E235Q at the end of 100ns MD run.

Furthermore, hydrogen bond (H-bond) analysis was performed for the native and mutant systems of FOXM1, which exhibited differences in the number of intermolecular H-bonded interactions among the individual systems. The average number of H-bonds ranged between 53.92 and 66.93 in the native and FOXM1 variants (Fig. 3e). All four shortlisted FOXM1 variants, viz. E235Q, R256C, S756P and G429E showed less number of H-bonds than the wild protein. A greater number of H-bonds are known to be associated with higher and enhanced system stability, as found in the native FOXM1 (66.93). Likewise, total energy was computed throughout 100ns simulation for the native and mutant systems of FOXM1 TF. The more negative the protein systems’ total energy value, the higher their corresponding conformational stability is. Figure 3f shows the combined total energy graphs of the native and mutant FOXM1 variants, with almost comparable values of fluctuating energies. The intrinsic statistics of dynamic stability of native and mutant systems of the FOXM1 TF have been summarized in **Supplementary Table 2**. The superimposed images of the native FOXM1 and its most deleterious variants E235Q and S756P at the 0ns and 100ns of MDS have been provided in Fig. 4.

### Molecular docking analysis of FOXM1 variants with DNA and protein-DNA contact analysis

**Fig. 5:**
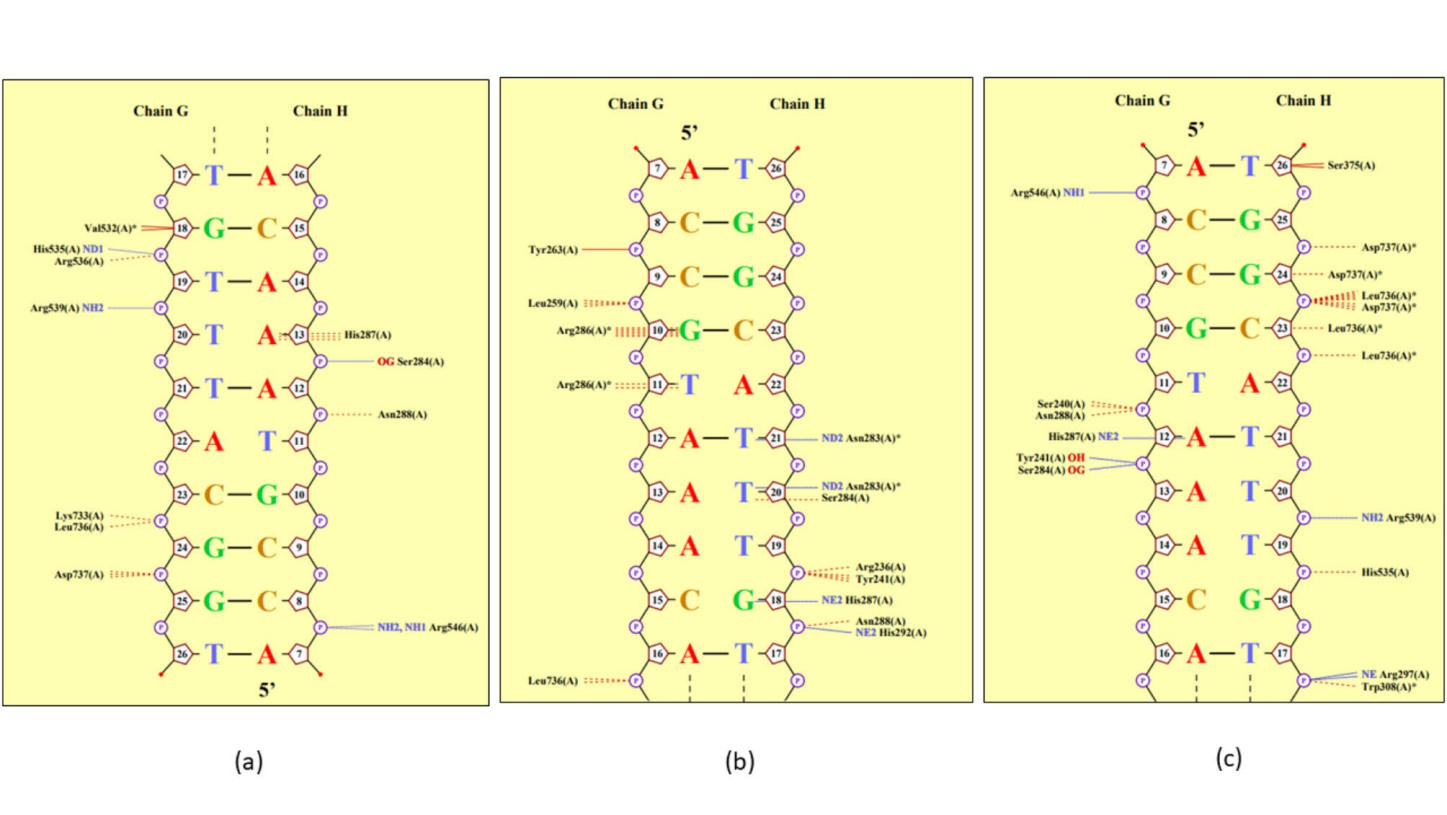
FOXM1-DNA binding at 100ns (a) Wild type (b)E235Q (c) S756P.

Molecular docking and protein-DNA contact analysis of native FOXM1 and its corresponding damaging variants with a 26 bp double-stranded DNA revealed that the mutants interacted with the consensus nucleic acid sequence in a slightly different orientation than the wild-type FOXM1 (Fig. 5). Upon DNA binding, the major interacting residues of native FOXM1 were Ser284, His287, Asn288, Val532, His535, Arg536, Arg539, Arg546, Lys733, Leu736 and Asp737. In contrast, the most obnoxious malignant variant E235Q predicted through our study was found to interact with the same nucleic acid sequence through residues Arg236, Tyr241, Tyr263, Leu259, Asn283, Ser284, Arg286 and His292. On the other hand, the major interacting residues, in the second-most predicted lethal mutant predicted from our study, S756P are Ser240, Tyr241, Ser284, His287, Asn288, Arg297, Trp308, Ser375, His535, Leu736 and Asp737. His535 and Asp73p were found to be among the common interacting residues in native FOXM1 and the S756P variant. Tyr241 was found to be a common interacting residue of both the mutants E235Q and S756P but absent in the native TF. Ser284, His287, Asn288, and Leu736, were observed to participate in DNA interaction in the wild and both the mutant variants of FOXM1. The interactions between the amino acid residues of the TF and the nitrogenous bases of the DNA denote the affinity of the native and mutant variants of FOXM1 to DNA binding. The change in the residue interaction occurring at the protein-DNA interface by the mutants E235Q and S756P is thus suggestive of their probable role in the structure destabilization of the native protein.

### Protein-drug molecular docking and RMSD calculation of FOXM1 variants

**Fig. 6:**
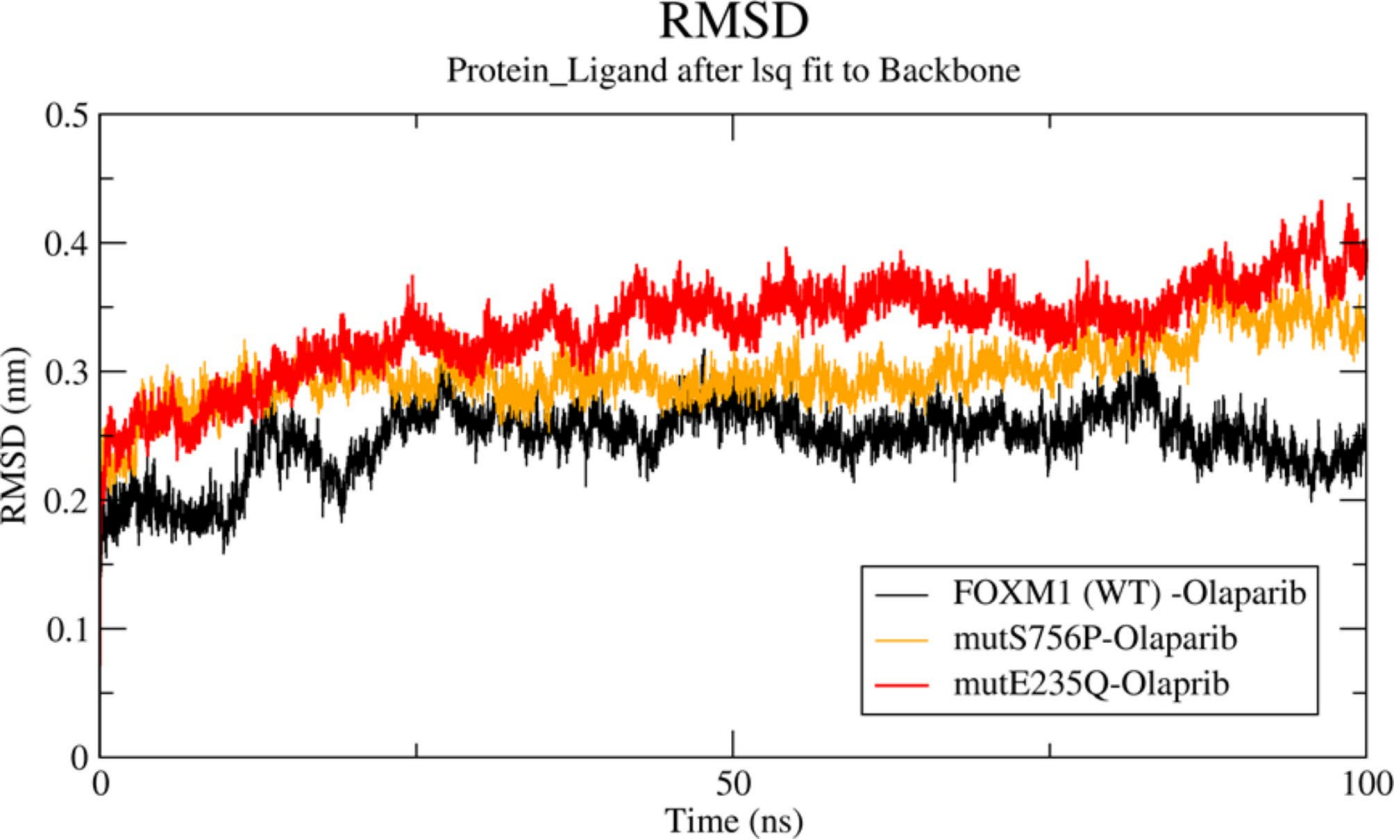
RMSD calculated over 100ns of wild-type FOXM1 and nsSNPs E235Q and S756P complexed with Olaparib.

Protein-ligand molecular docking using AutoDockTools 1.5.6 was performed for the tertiary structures of the modeled protein (native and mutant) of FOXM1 TF, with an FDA-approved clinically standardized drug for TNBC, Olaparib. The binding association of the FOXM1 mutational variants with the Olaparib was found to vary from that of the native structure of the TF. The binding energies of the native and variant structures of FOXM1 with Olaparib were expressed in kcal/mol considering minimal H-bond distance between the catalytic active site residues of the native and mutant variants of FOXM1. The highest binding affinity with the least binding energy score of -7.5 kcal/mol was found for the native FOXM1-Olaparib complex. On the contrary, the least binding affinity with the highest binding energy score of -6.5 kcal/mol was found in the mutant E235Q, followed by the variant S756P with an intermediate binding energy score of -7 kcal/mol. The binding energy scores obtained from molecular docking were further confirmed by calculating the RMSD values of the three protein-ligand complexes executed over 100 ns simulations. The lower the RMSD value, the higher the ligand-bound complex’s stability. In concordance with the results of the binding energy scores, as observed from the combined RMSD graphs plotted for the three complexes (Fig. 6) the mean least RMSD value of the native FOXM1-Olaparib complex was observed to be 0.246 nm (denoted in black), whereas the highest mean RMSD of 0.334 nm was calculated for the variant E235Q-Olaparib complex (denoted in red). The binding energy scores, key interacting residues, minimum H-bond distances, and the corresponding values of RMSD of the native and mutant variants of FOXM1 have been provided in Supplementary Table 3.

## Discussion

Owing to its extreme molecular heterogeneity, each subtype of BC differs in its histopathological features, molecular characteristics, therapy response and prognosis^71^. Member of the Forkhead/winged helix superfamily of TFs, the overexpression of this proliferation-promoting oncogenic TF was highest in both the primary and recurrent tumors of the most aggressive BC subtype, TNBC^23^. Despite identifying multiple molecular hallmarks of TNBC, the FOXM1-driven TNBC molecular invasion is not completely elucidated. In addition to tumor cell proliferation, the role of FOXM1 has been reported in several cellular processes including, inflammation, apoptosis hindrance, angiogenesis and metastasis promotion, therapy resistance, renewal of stem cells and maintenance of mitotic spindle integrity^20^.

SNPs are the most common and highest-occurring genetic variations in the human genome associated with enhanced malignant susceptibility^30,31^. Comprehending the molecular etiology of diverse cancers including sporadic tumors such that of TNBC necessitates a thorough understanding of the processes that underlie the impact of SNPs that contribute to cancer susceptibility^72^. Hence from a clinical perspective, SNPs serve as potential therapeutic and diagnostic biomarker tools that aid in the understanding of the molecular vulnerabilities that drive cancer progression and prognosis. Due to the implementation of several high-throughput sequencing techniques, there is an oversaturated surge in the number of known nsSNPs. Characterizing these known non-synonymous variants is often an expensive, laborious and time-consuming endeavor regarding their relative contribution to a particular phenotype^73^. Additionally, the exponential increase in the nsSNP numbers makes it inconvenient to ascertain their biological impact through laboratory research. In contrast, using computational in-silico techniques to differentiate the pathogenic disease-causing variants from the neutral SNPs retrieved from a huge pool of SNP datasets may greatly accelerate the process. Of late, multiple experimental investigations have been conducted to evaluate the link between nsSNPs and drug therapy in the context of cancer treatment. Several researchers and scientists have already delineated and reported the involvement of the pathogenic nsSNP variants on protein folding, stability, structural and functional alteration, conservation, post-translational modifications, DNA and ligand binding, which might cause or affect malignancy prognosis^74^. Likewise, huge number of nsSNPs have been detected for the oncogenic FOXM1 TF. However, not every SNP contributes to conformational and functional modification of the protein or might produce a consequence of minimum frequency making it infeasible for analysis^75^. Hence, using various computational tools and techniques, the current in-silico study have aimed at the identification of novel pathogenic malignant nsSNPs of FOXM1 TF, that may affect TNBC progression and prognosis.

We have used seven different computational webservers notably SIFT, PANTHER-PSEP, SNAP, PolyPhen-2, PON-P2, PhD-SNP and SNPs&GO for the identification of damaging, malignant and disease-associated SNPs from the huge pool of SNP datasets retrieved from NCBI dbSNP (1st phase filtration). Each amino acid substitution in the native protein, may impact protein function. SIFT help users prioritize amino acid substitutions retrieved from a huge dataset of SNPs which are anticipated to affect protein function. Ranging from 0 to 1, any substitution with a score higher than 0.5 is considered to be malignant^2^. On the otherhand, PANTHER-PSEP employs an evolutionary preservation-based metric algorithm for the prediction of potentially pathogenic nsSNPs. As mentioned earlier, a high confidence score on the scale of 0 to 1, implies detrimental effects of the substitution on the protein function^3^. Contrastingly, SNAP is a neural-network derived algorithm that facilitates protein function prediction by incorporating additional information such as secondary structure of proteins, evolutionary information of conserved residues of proteins, solvent accessibility and other relevant informations. A machine-learning based algorithm PON-P2, is another protein function predicting algorithm, where a score of 0.9-1 is interpreted to be highly pathogenic, an intermediate range between 0.5 and 0.89 signifies moderate confidence and possible pathogenicity, and lastly, a low confidence score of less than 0.5 corresponds to benign variation with least pathogenicity. Using conformational and relative evolutionary considerations, PolyPhen-2 (Polymorphism Phenotyping v2), forecasts the potential effects of amino acid alterations on the stability and functionality of human proteins. SNPs are functionally annotated, coding SNPs are mapped to gene transcripts, protein sequence annotations and structural characteristics are extracted, and conservation profiles are constructed. Based on a combination of all these characteristics, it then calculates the likelihood that the missense mutation would be harmful. On a scale of 0 to 1, a high PolyPhen-2 score of greater than 0.9 is considered as “probably damaging”^4^. Another webserver tool, PhD-SNP categorizes the substitution variations into benign and pathogenic based on a confidence score ranged between 0 and 1. Any substitution variation with a score ≥ 0.5 is considered to be pathogenic^5^. And conclusively, the final webserver used in thi9s study for predicting nsSNP pathogenicity, SNPs&GO uses functional annotations for SNP classifications based on RI scoring system ranged between 0 (neutral effect on protein function) to 1 (deleterious effect on protein function)^6^.

Since the algorithms employed by these seven webservers were theoretically distinct and unique thus good prediction reliability would be guaranteed by the confidence score obtained from all those servers. Ultimately, four nsSNPs viz. E235Q, R256C, G429E and S756P were commonly identified from 38 disease-causing pathogenic deleterious nsSNPs of FOXM1 protein which were then subjected to further downstream filtration analysis.

Identification of conserved residues of a protein are of crucial importance as they governs protein folding and stability. We have used the ConSurf tool to obtain the evolutionary conservation profile of the constituent amino acid residues of FOXM1 TF. ConSurf analysis confirmed all four nsSNPs identified as the most damaging malignant nsSNPs of FOXM1 were highly conserved, with variants E235Q and S756P having buried and G429E and R256C with exposed residues possibly located at the site of enzymatic interaction of the protein thus participating in significant protein-protein interactions .Additionally, MusiteDeep a deep-learning based algorithm was used to predict the role of the shortlisted nsSNPs in the PTM site modification of the FOXM1 protein. The stability of the nsSNPs were further evaluated using webservers Mupro and I-mutant tools, which characterized the final phase of our second-phase filtration analysis.

After shortlisting the nsSNPs from the 1st and 2nd filtration analysis, three-dimensional structures of the four nsSNP variants and the native protein were designed to further comprehend the structure level modifications and alterations that might be attributable to these detrimental polymorphic shifts. Hence different MD simulation analyses were performed using the GROMACS software, version 2023.1 for each of the variant and native tertiary structure of the FOXM1 protein, executed over 100ns to evaluate the behavior of the modeled variants in a simulated environment. The mean average RMSD, RMSF, Rg, SASA was found to be the least in the native wild-type modeled structure of the FOXM1 TF, indicating high stability, less perturbations and conformational transitions compared to the altered structures of the variants. The increased mean RMSD value of the variants exhibited the unstable nature of the mutants. Similarly, higher value of mean RMSF implicated enhanced backbone flexibility of the four most damaging nsSNPs shortlisted through our study. The variant G429E showed enhanced conformational compactness with a mean Rg value of 1.23707 nm compared to the other three FOXM1 variants. Enhanced conformational expansion was observed in the variant E235Q with the highest average Rg of 1.66501 nm. The four nsSNP variants exhibited substantial differences in their respective mean SASA values compared to the wild-type native FOXM1 tertiary structure possible contributing to changes in the protein-protein interactions.The number of intrinsic hydrogen interactions which denotes system stability was also found to be higher in the wild FOXM1 with less total interaction energy expressed in kcal/mol compared to the mutants viz. E235Q, R256C, G429E and S756P. As implicated from the different MD analyses, the structural assaults due to the missense polymorphisms was found to be most pronounced and detrimental in the mutants, E235Q followed by S756P among the four most damaging variants. Therefore, during aggregation and folding, these aberrant alterations these aberrant alterations of the amino acid residues at positions 235 and 756 of the FOXM1 TF might have caused an immense loss of thermodynamic stability. Therefore, based on the inferences of the MDS analyses, it is thus hypothesized that the variants viz. E235Q, S756P, R256C and G429E are anticipated to have a detrimental effect on the structural and functional attributes of the FOXM1 protein since they have been shown to produce alterations in the inherent conformation of the protein in certain behaviors. Additionally, protein-DNA contact analysis using NUCPLOT revealed that the DNA interacting residues varied in between the native and variant forms of the FOXM1 TF. Most of the protein-DNA interactions were observed between the positively charged amino acid residues and the negative phosphodiester bond of the DNA. The greater the number of protein-DNA contacts higher is the binding affinity. Since the protein-DNA interactions are majorly stabilized by H-bonds, the prevalence of less number of H-bonds in the variants as compared to that found in the native model of the protein, are known to affect the interaction of the variants with the consensus DNA of the TF. Further molecular docking of the native and the two most damaging mutants as identified from MDS studies, viz. E235Q and S756P with an FDA-approved TNBC inhibitor, Olaparib revealed. As inferred from the scores of their respective binding energies, the mutant E235Q followed by S756P exhibited a weaker binding motif with Olaparib than the native tertiary structure of FOXM1. Major disruptive binding perturbations of Olaparib with catalytic binding pocket of the mutants E235Q and S756P were also further validated from the higher value of mean RMSD of the variant nsSNP FOXM1-Olaparib complexes compared to the native protein indicative of increased receptor-drug complex incompatibility.

## Conclusion

The strong and highest overexpression of the FOXM1 TF approximately in 85% of TNBC patients across all BC subtypes makes it a lucrative biomarker in decoding both TNBC therapeutics and prognosis. Despite the elevated overexpression of the oncogenic TF FOXM1 in TNBC which is associated with an extremely poor prognosis of the disease and relatively shorter OS, FOXM1-mediated TNBC pathogenesis is still not completely elucidated. This concomitant sequence-based in-silico and structural investigation has identified the deleterious FOXM1 nsSNPs and their impact on stability, evolutionary conservation, role in PTM, structure and function of the protein. Additionally, the interaction of the strongly overexpressed oncogenic TNBC TF with the DNA and their respective ligand binding sites is also evaluated. E235Q was identified as the most damaging malignant mutant of FOXM1, among the four pathogenic mutants viz. E235Q, S756P, G429E, and R256C which were shortlisted in our study from a huge dataset pool of 8826 SNPs. Although, further in-vitro validations are required to confirm these computational findings still the in-silico shortlisting of the damaging and malignant nsSNPs will eventually aid in shortening the time-consuming, expensive and laborious in-vitro protocol of SNP identification followed in wet-lab studies. Concisely, the findings of this investigation could be used as a blueprint for malignant nsSNP identification of FOXM1 TF aiding in clinical TNBC therapeutics.

## Electronic supplementary material

Below is the link to the electronic supplementary material.

Supplementary Material 1

## Data Availability

All datasets generated and/or analysed during this study are included in this published article [and its supplementary information files].
