# Supplementary material for: A computational and structural approach to identify malignant non-synonymous FOXM1 single nucleotide polymorphisms in triple-negative breast cancer
Source: Sci Rep. 2025 Jan 6;15:964. doi: 10.1038/s41598-024-85100-w (PMC11704209; doi:10.1038/s41598-024-85100-w)
Supplement: Supplementary file 1 — Supplementary Material 1 [file 41598_2024_85100_MOESM1_ESM.pdf]

A computational and structural approach to identify malignant non-synonymous FOXM1 single nucleotide polymorphisms in Triple-negative Breast Cancer

*Prarthana Chatterjee[a], and Satarupa Banerjee**

[a]School of BioSciences and Technology, Vellore Institute of Technology, Vellore- 632014, Tamil Nadu, India.

[a*]Corresponding author: Assistant Professor, School of BioSciences and Technology, Vellore Institute of Technology, Vellore- 632014, Tamil Nadu, India. Email id: satarupa.banerjee@vit.ac.in, satarupabando@gmail.com

Supplementary Table 1

rsID	Substitution position	Deleterious mutation probability	Structural and functional impact
rs776324631	E235Q	0.792	Altered ordered interface (P=0.05),<br>Altered DNA binding (P= 5.2e-03),<br>Loss of allosteric site at E97 (P=6.4e-03),<br>Loss of methylation at K96 (P= 9.7e-04),<br>Gain of the catalytic site at K96 (P= 0.02),<br>Altered stability at E97 (P= 0.05)
rs137928577	R256C	0.561	Loss of N-linked glycosylation at N715 (P = 0.04),<br>Loss of intrinsic disorder (P= 0.04)
rs1285576061	G429E	0.606	Altered transmembrane protein (P= 1.2e-04),<br>Altered metal binding (P= 1.0e-03),<br>Altered disorganized interface (P= 0.03),<br>Gain of the loop (P= 0.03)<br>Gain of relative solvent accessibility (P=0.04)

			Altered stability (P= 0.05)
rs1216720660	S756P	0.739	Altered disordered interface (P=0.05),<br>Loss of allosteric site at W755 (P= 0.03),<br>Loss of N-linked glycosylation at N754 (P = 0.04)

Table 1 Structural and functional impact of pathogenic diseased nsSNPs on FOXM1.

Supplementary Table 2

**Protein/ mutant system**	**RMSD (nm)**		**RMSF (nm)**		**Rg (nm)**		**SASA(nm^2)**		**H-bond numbers**	
	**Range**	**Average**	**Range**	**Average**	**Range**	**Average**	**Range**	**Average**	**Range**	**Average**
Wild-type FOXM1	0.0502954-3.5657861	3.103631201	0.2801-2.4824	1.138423853	3.4348-5.43344	3.945755916	550.3-968.534	646.1152681	23-93	66.93130687
R256C	0.0494665-3.7654357	3.231344856	0.3584-3.1426	1.237076016	3.63194-5.43591	4.081174603	574.054-967.501	662.9228972	26-84	58.12177006
G429E	0.0499448-3.6469874	3.24782869	0.4501-2.6821	1.22249882	3.45454-5.43583	4.022699208	577.274-971.063	653.7806756	23-89	61.02709729
E235Q	0.0460894-4.4071841	3.64377596	0.4591-3.1886	1.665012975	3.80517-5.4345	4.456375063	572.904-971.477	670.1707331	25-78	53.92840716
S756P	0.0485786-4.1473312	3.572089645	0.6092.70652-	1.464071822	3.8676-5.53642	4.3373314	555.192-968.357	667.0666299	24-89	57.99350779

Table 2 depicts the average RMSD, RMSF, Rg, SASA and H-bond values for FOXM1 and its mutants viz. R256C, E235Q, G429E and S756P.

Supplementary Table 3

**FOXM1-drug models**	**Binding energy (Kcal/mol)**	**Contact residues**	**Average RMSD (nm)**
**Wild type-Olaparib**	**-7.5**	**Met257, Thr258 Leu259, Lys260 Asp261, Tyr263, His287 and Leu289**	**0.24684**
**mutE235Q-Olaparib**	**-6.5**	**Ser290, Arg297 Thr299, Lys304 Val305, Ser306 ,Phe307 and Trp308**	**0.33401**
**mutS756P-Olaparib**	**-7**	**Arg286, His287 , Leu289 Ser290 LEU291,Phe295 VAal296, Asp648, Pro651 and Asp658**	**0.28684**

Table 3 AutoDock calculated binding interaction results of wild type FOXM1 alongwith its most damaging nsSNPs viz. mutE235Q and mutS756P depicting major interacting residues and average RMSD.

Supplementary Fig.1.1

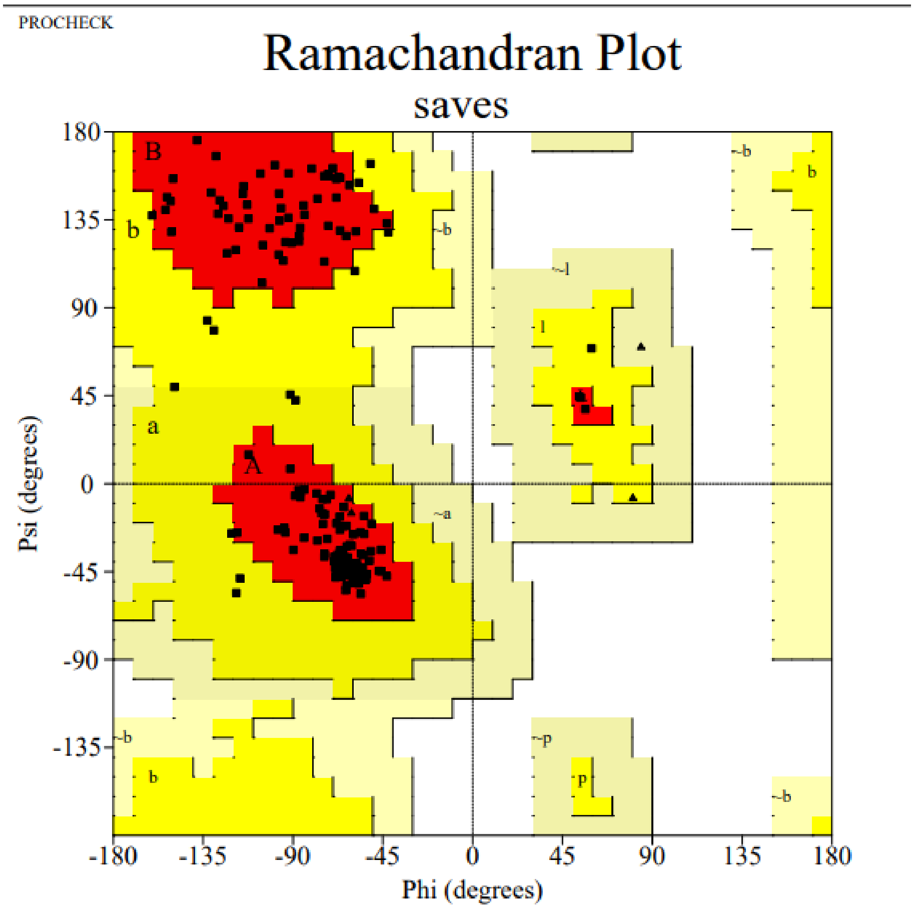

Plot statistics

Residues in most favoured regions [A,B,L]	153	92.2%
Residues in additional allowed regions [a,b,l,p]	13	7.8%
Residues in generously allowed regions [~a,~b,~l,~p]	0	0.0%
Residues in disallowed regions	0	0.0%
	----	------
Number of non-glycine and non-proline residues	166	100.0%
Number of end-residues (excl. Gly and Pro)	46	
Number of glycine residues (shown as triangles)	4	
Number of proline residues	11	

Ramachandran plot analysis of native FOXM1

Fig1.2

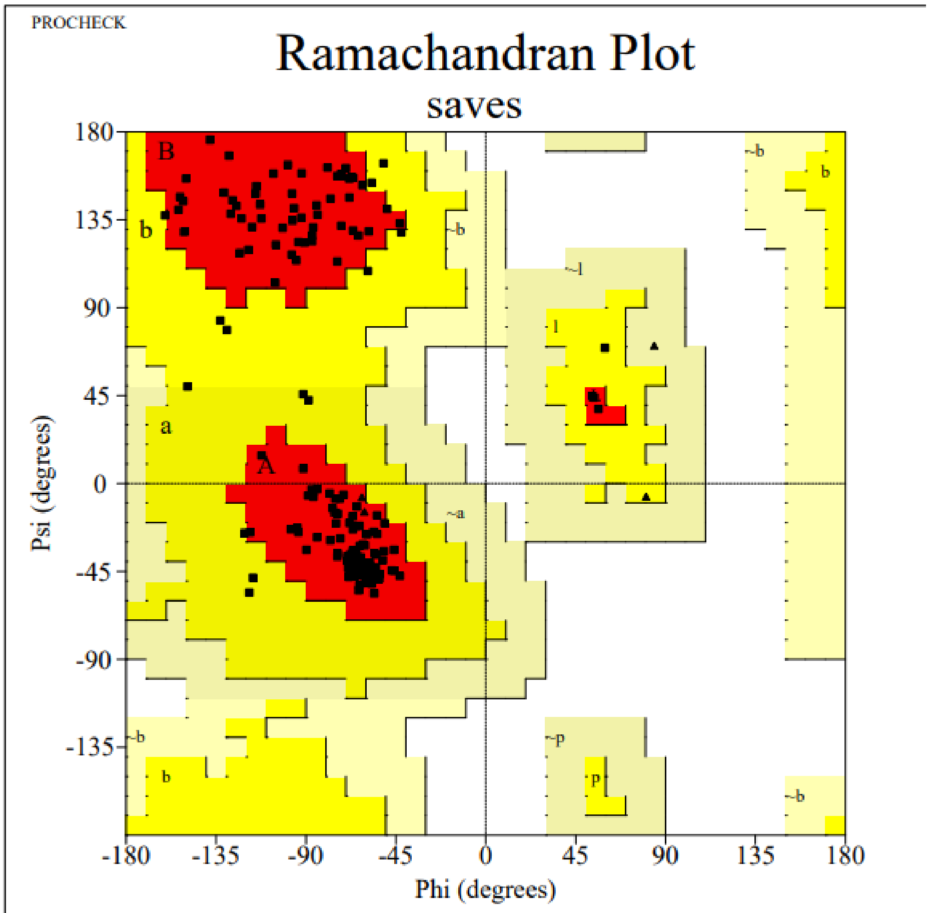

Plot statistics

Residues in most favoured regions [A,B,L]	153	92.2%
Residues in additional allowed regions [a,b,l,p]	13	7.8%
Residues in generously allowed regions [~a,~b,~l,~p]	0	0.0%
Residues in disallowed regions	0	0.0%
	----	------
Number of non-glycine and non-proline residues	166	100.0%
Number of end-residues (excl. Gly and Pro)	48	
Number of glycine residues (shown as triangles)	4	
Number of proline residues	11	

Ramachandran plot analysis of FOXM1 mutant, E235Q.

Fig1.3

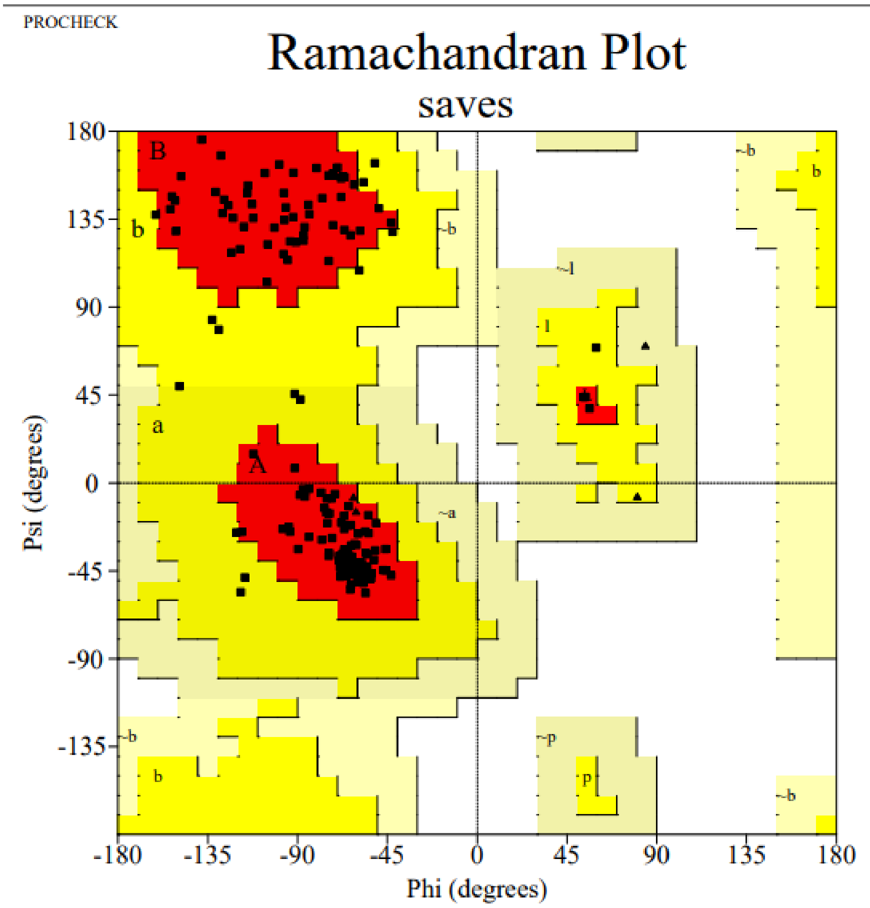

Plot statistics

Residues in most favoured regions [A,B,L]	153	92.2%
Residues in additional allowed regions [a,b,l,p]	13	7.8%
Residues in generously allowed regions [~a,~b,~l,~p]	0	0.0%
Residues in disallowed regions	0	0.0%
	----	------
Number of non-glycine and non-proline residues	166	100.0%
Number of end-residues (excl. Gly and Pro)	48	
Number of glycine residues (shown as triangles)	4	
Number of proline residues	11	

Ramachandran plot analysis of FOXM1 mutant, R256C.

Fig.1.4

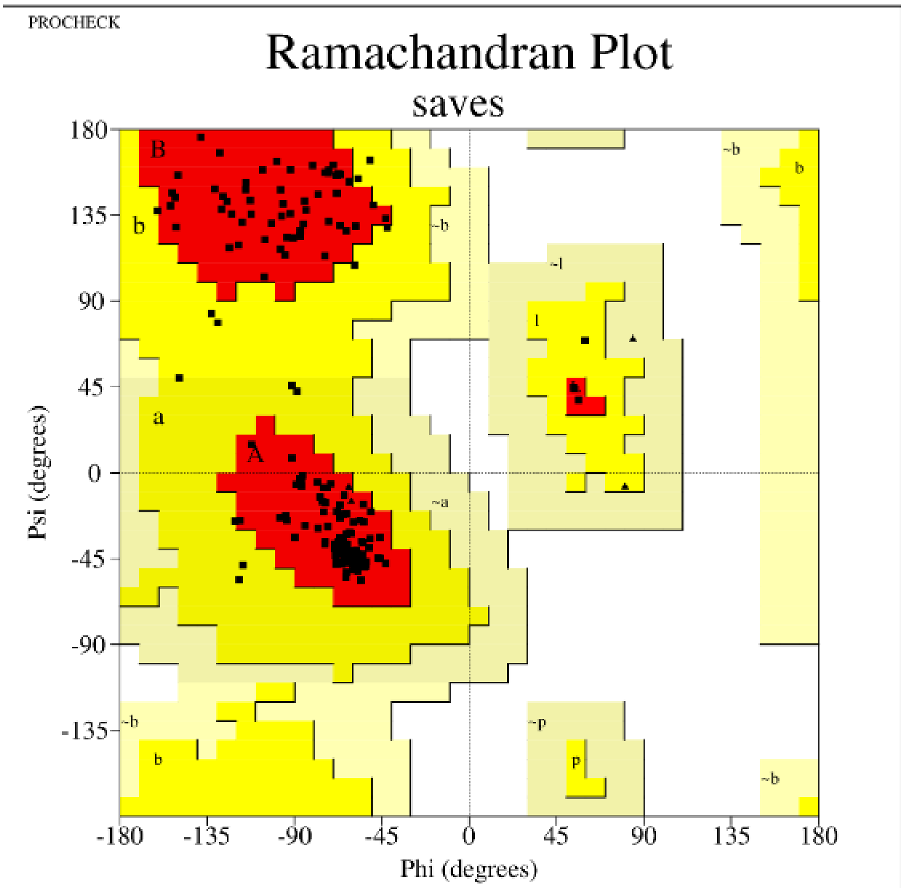

Plot statistics

Residues in most favoured regions [A,B,L]	153	92.2%
Residues in additional allowed regions [a,b,l,p]	13	7.8%
Residues in generously allowed regions [~a,~b,~l,~p]	0	0.0%
Residues in disallowed regions	0	0.0%
	----	------
Number of non-glycine and non-proline residues	166	100.0%
Number of end-residues (excl. Gly and Pro)	48	
Number of glycine residues (shown as triangles)	4	
Number of proline residues	11	

Ramachandran plot analysis of FOXM1 mutant, G429E.

Fig. 1.5

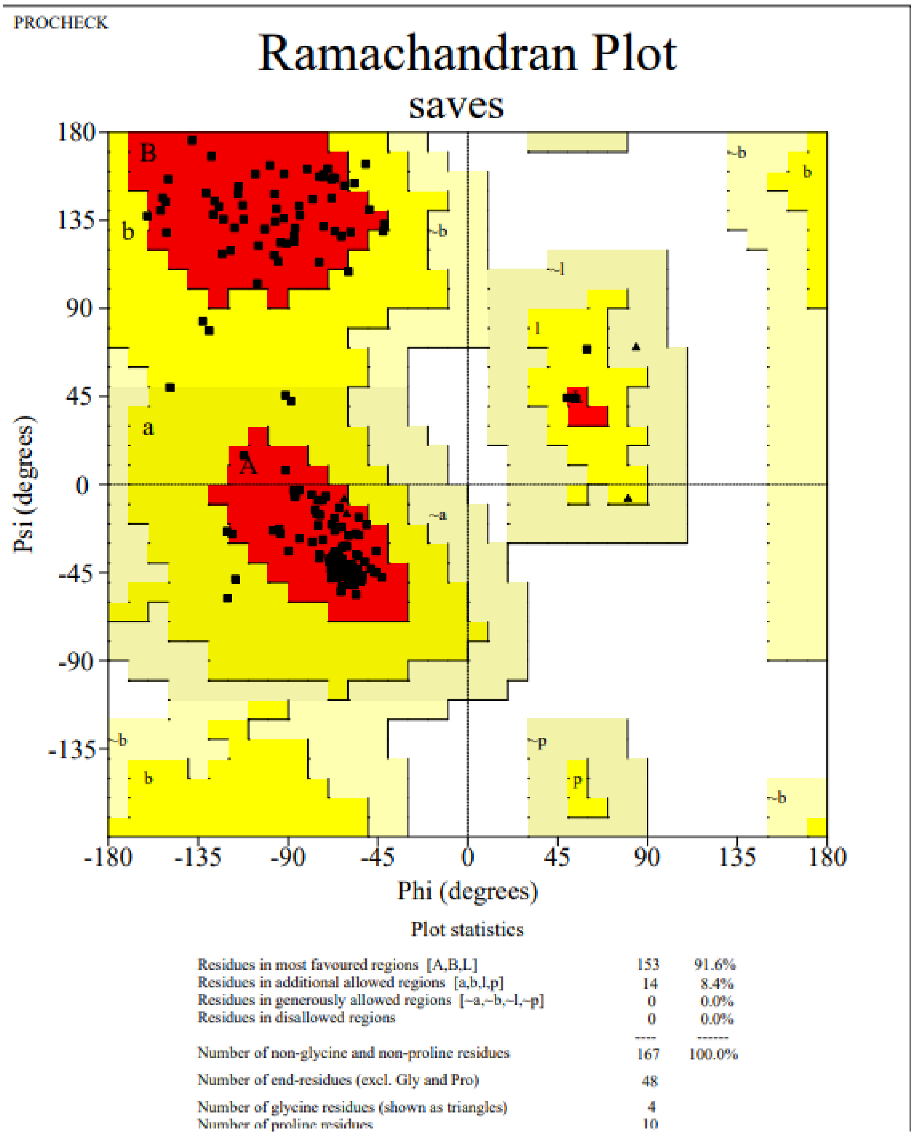

Ramachandran plot analysis of FOXM1 mutant, S756P.

Supplementary Fig.2

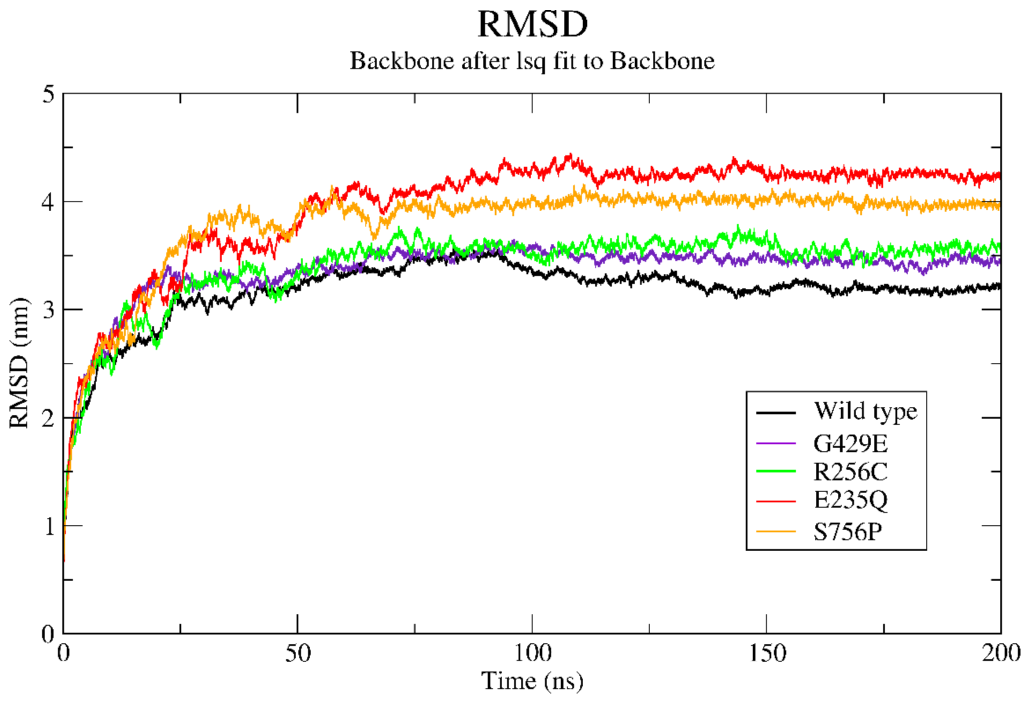

Fig.2 Combined RMSD graph extended over 200ns of the mutants G429E, R256C, E235Q and S756P, along with the wild-type FOXM1 TF.
